# In vivo magnetic particle imaging: angiography of inferior vena cava and aorta in rats using newly developed multicore particles

**DOI:** 10.1038/s41598-020-74151-4

**Published:** 2020-10-14

**Authors:** Azadeh Mohtashamdolatshahi, Harald Kratz, Olaf Kosch, Ralf Hauptmann, Nicola Stolzenburg, Frank Wiekhorst, Ingolf Sack, Bernd Hamm, Matthias Taupitz, Jörg Schnorr

**Affiliations:** 1 Experimental Radiology, Department of Radiology, Charité-Universitätsmedizin Berlin, corporate member of Freie Universität Berlin, Humboldt-Universität zu Berlin, Berlin Institute of Health, Chariteplatz 1, 10117 Berlin, Germany; 2 Department of Medical Physics and Metrological Information Technology, Physikalisch-Technische Bundesanstalt (PTB), Abbestrasse 2-12, 10587 Berlin, Germany

**Keywords:** Three-dimensional imaging, Imaging techniques and agents, Medical imaging

## Abstract

Magnetic Particle Imaging (MPI) is a new imaging modality, which maps the distribution of magnetic nanoparticles (MNP) in 3D with high temporal resolution. It thus may be suited for cardiovascular imaging. Its sensitivity and spatial resolution critically depend on the magnetic properties of MNP. Therefore, we used novel multicore nanoparticles (MCP 3) for in-vivo MPI in rats and analyzed dose requirements, sensitivity and detail resolution. 8 rats were examined using a preclinical MPI scanner (Bruker Biospin GmbH, Germany) equipped with a separate receive coil. MCP 3 and Resovist were administered intravenously (i.v.) into the rats’ tail veins at doses of 0.1, 0.05 and 0.025 mmol Fe/kg followed by serial MPI acquisition with a temporal resolution of 46 volumes per second. Based on a qualitative visual scoring system MCP 3–MPI images showed a significantly (P ≤ 0.05) higher image quality than Resovist-MPI images. Morphological features such as vessel lumen diameters (D_*L*_) of the inferior vena cava (IVC) and abdominal aorta (AA) could be assessed along a 2-cm segment in mesenteric area only after administration of MCP 3 at dosages of 0.1, 0.05 mmol Fe/kg. The mean D_L_ ± SD estimated was 2.7 ± 0.6 mm for IVC and 2.4 ± 0.7 mm for AA. Evaluation of D_*L*_ of the IVC and AA was not possible in Resovist-MPI images. Our results show, that MCP 3 provide better image quality at a lower dosage than Resovist. MCP 3-MPI with a clinically acceptable dose of 0.05 mmol Fe/kg increased the visibility of vessel lumens compared to Resovist-based MPI towards possible detection of vascular abnormalities such as stenosis or aneurysms, in vivo.

## Introduction

Magnetic particle imaging (MPI) was first introduced in 2005 by Gleich and Weizenecker^1^. MPI is a noninvasive, tomographic imaging modality that maps the 3D-distribution of magnetic nanoparticles (MNP) giving quantitative information on the local MNP concentrations. MPI furthermore allows for a high temporal resolution with 46 3D frames per second (f/s) at a spatial resolution in the millimeter range. MPI images display only the distribution of the MNP and do not contain morphologic background information. Thus, qualitatively, MPI images can be compared with tomographic radiotracer images, as e.g. obtained with Single Photon Emission Computed Tomography (SPECT). The detection of MNP is based on the nonlinear magnetization response of the particles when exposed to an oscillating external magnetic field^2^. MPI is commonly performed using iron oxide nanoparticles (Magnetite/Maghemite) with appropriate magnetic characteristics for MPI. The resolution of MPI images is determined by a combination of MPI scanner properties such as the power and slew rate of the magnetic field gradients, as well as the MNP used.

With further advances in MPI, a number of in vitro and in vivo studies have provided the proof of concept of cardiovascular imaging. The first in vivo MPI images were obtained in 2009 and visualized the circulatory system and the beating heart of a mouse^2^. The images depicted the large vessels including the vena cava and the cardiac chambers. Since then the heart and vena cava of mice have been repeatedly scanned with various MPI systems incorporating advances in MPI technology, such as X-space MPI^3^, traveling wave MPI (TWMPI)^4^, and the use of sophisticated separate receive coils^5,6^ or in tracer development such as LS-008 particles^3,7^. These studies have shown improvements in angiographic image quality by improving sensitivity and resolution. Phantom studies show that MPI allows quantitative visualization of vascular abnormalities such as stenosis^8,9^ and aneurysm^10,11^ or that MPI can be used to track MNP-labeled material for vascular interventions like balloon catheters^12,13^.

This study introduces newly developed multicore nanoparticle (MCP 3)^14^ as tracers for cardiovascular MPI. To this end, we administered the new tracers into the tail vein of rats and then performed imaging in a commercial in vivo MPI system. The sensitivity of MPI for detecting MCP 3 was compared with that of conventional tracers (Resovist). Finally, we evaluated the capability of MCP 3-based MPI to assess in vivo anatomical features of the abdominal aorta (AA) and the inferior vena cava (IVC).

## Results

**Figure 1:**
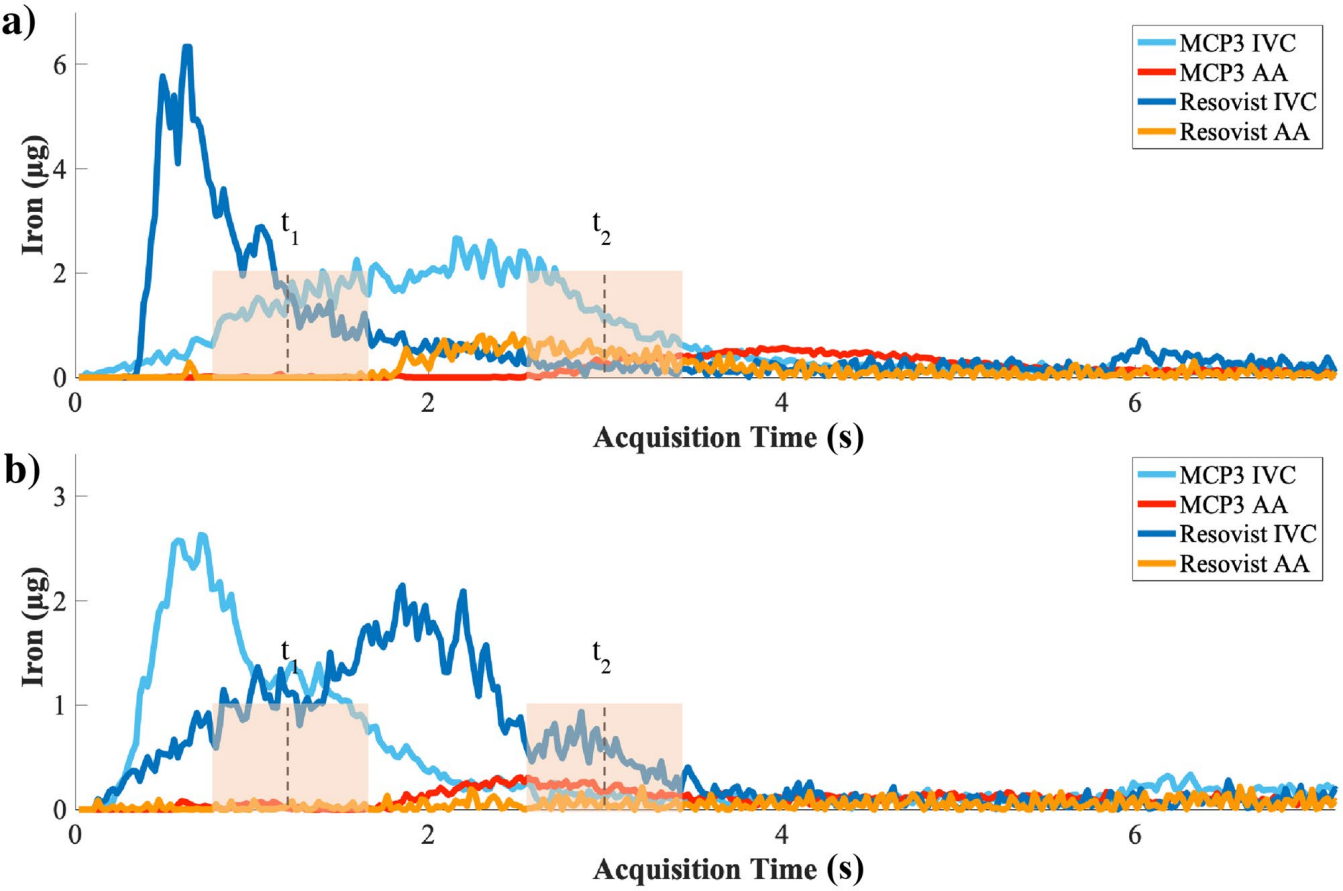
Iron concentration–time curves of the IVC and first-pass in the AA for both tracers, MCP 3 and Resovist, in the VOI defined in imaging data acquired. Shown are curves for administered doses of 0.1 mmol Fe/kg **(a)** and 0.05 mmol Fe/kg **(b)**. The iron level of Resovist-based arterial phase in **(b)** is hardly distinguishable from noise. t_1_ and t_2_ indicate the time points when bolus in IVC and arterial phase in AA were further analysed.

The temporal profiles of the iron concentration in the volume of interest (VOI) over IVC and AA are presented in Fig. 1. The propagation of bolus into the IVC (t_1_—time point 1–1.5 s after injection) and arterial phase (t_2_—approx. 2–3 s after initial MNP administration) is seen in the signal rise in iron concentration–time curves. It takes approx. 2 s for the bolus to pass from the site of i.v. injection until reaching the AA. The profiles of iron concentrations over time look different due to different injection speed as the injections were performed manually. The t_1_ and t_2_ time points were chosen accordingly based on visual assessment of reconstructed images, assuring uniform bolus distribution without any impact from manual injection. Images of IVC reconstructed at the time of peak of bolus or nearly after (t_1_) showed similar adequate image quality (Supplement S1) except for Resovist at dosage of 0.025 mmol Fe/Kg, where the IVC image provided adequate image quality only at time of peak of bolus. The contributing factors in the image quality in images of MCP 3 in comparison to Resovist are the higher signal amplitude and slower drop of higher harmonics (Supplement S2)^14^.

**Figure 2:**
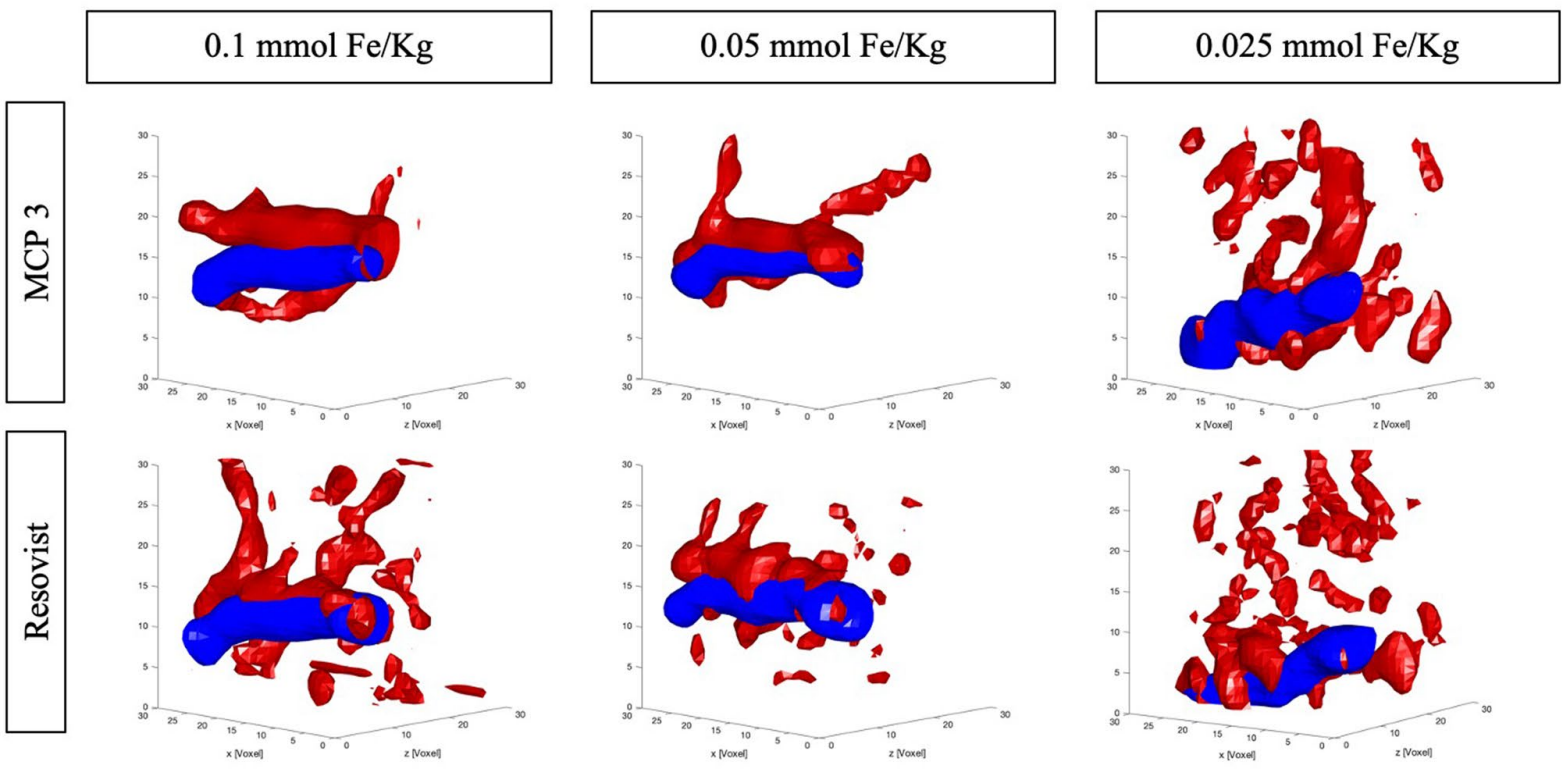
In vivo MPI images of the IVC (blue) and arterial phase (red) in rats. Arterial phase is clearly visible without degradation by artifacts with higher dosages of MCP 3. Conversely, arterial phase is hardly detectable with Resovist, and the images are characterized by an overall lower image quality and more severe background noise artifacts.

**Figure 3:**
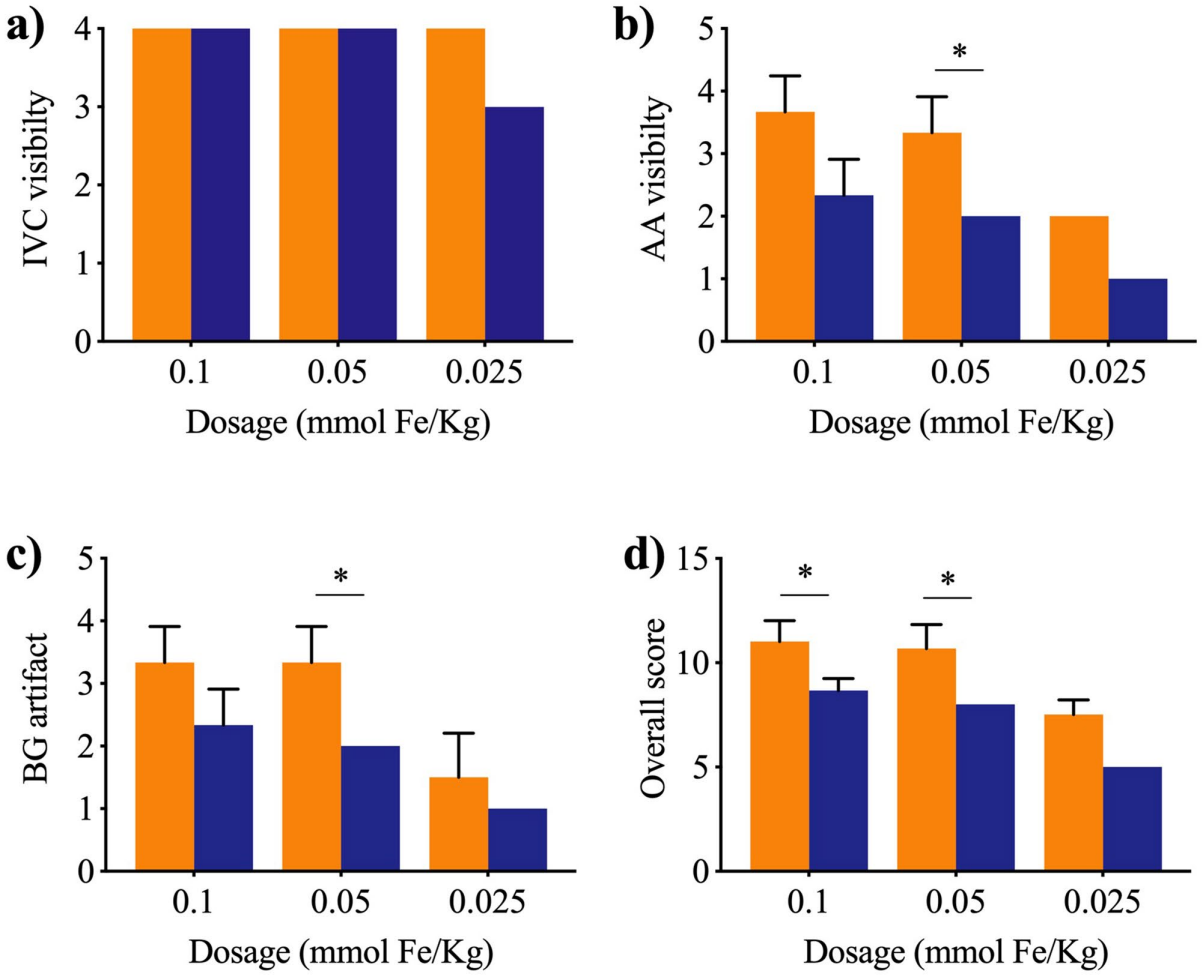
Mean VGAS as assessed on a 4-point scale at all dosages based on average score. MCP 3 (orange) and Resovist (purple). MPI images of MCP 3 show a higher VGAS in all image criteria, characterized by an overall higher image quality. n = 3 for 0.1 and 0.05 mmol Fe/kg, n = 2 for 0.025 mmol Fe/kg. Data were compared using one-tailed Mann–Whitney U test (*P ≤ 0.05).

Examples of overlaid reconstructed images from two time points of t_1_ and t_2_ are presented in Fig. 2. The qualitative image analysis, shows a significant increase in MPI image quality with MCP 3 rather than Resovist. A detailed overview is reported in Fig. 3. Propagation of the bolus through the IVC was visualized clearly with all three doses of the two MNP however the arterial phase was discerned only with MCP 3 particles at administered dosages of 0.1 and 0.05 mmol Fe/kg with good adequate morphological information of AA. Minor artifacts were present in images acquired after MCP 3 administration, while more severe artifacts were apparent in images obtained with administration of Resovist.

**Figure 4:**
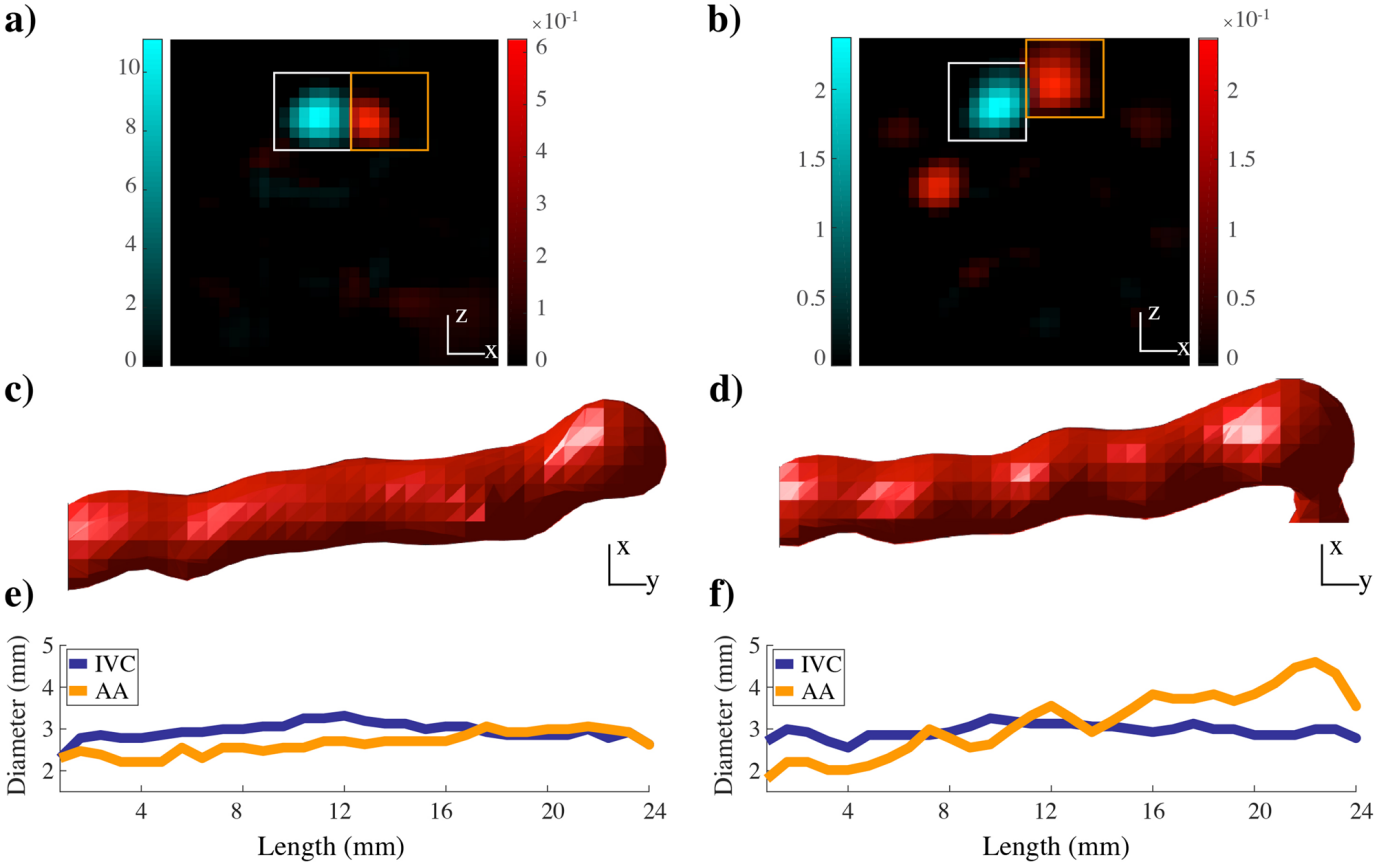
MPI digital subtraction angiography. Shown are the results for MCP 3 at 0.1 mmol Fe/kg in transverse **(a)** and coronal orientation **(c)** and the D_L_ **(e)** versus the corresponding results for 0.05 mmol Fe/kg **(b, d, f)**. Mean D_L_ is in the range of 2–3 mm. The white square in A and B indicates the IVC VOI and the orange square the AA VOI. The arterial phase is isolated by subtraction of the IVC during the bolus passage through AA. The D_L_ of the AA and abdominal IVC along their longitudinal axes is determined. Color bar values in **(a)** and **(b)** are in µg.

Resolution of MPI is in the millimeter range, which does not allow clear differentiation of the adjacent IVC and AA. To clearly visualize the AA during arterial phase and quantitatively estimate lumen diameters (D_*L*_)_*,*_ a digital subtraction step was carried out during and after bolus passage in the IVC (Fig. 4). The term digital subtraction refers to subtraction of two temporally separated images, an early post-contrast image (t_1_) is subtracted from a late post-contrast image (t_2_). The subtracted images allowed clear separation and independent assessment of the two adjacent vessels. Quantification of the D_*L*_ of vessels was feasible at higher concentrations of MCP 3 (0.1 and 0.05 mmol Fe/kg). Mean D_*L*_ was calculated along the longitudinal axis of the AA and IVC (Fig. 4). Mean D_L_ ± SD were as follows: (A) 0.1 mmol Fe/kg: IVC = 2.7 ± 0.7, AA = 2.5 ± 0.7; (B) 0.05 mmol Fe/kg: IVC = 2.8 ± 0.6, AA = 2.3 ± 0.8 in mm.

**Figure 5:**
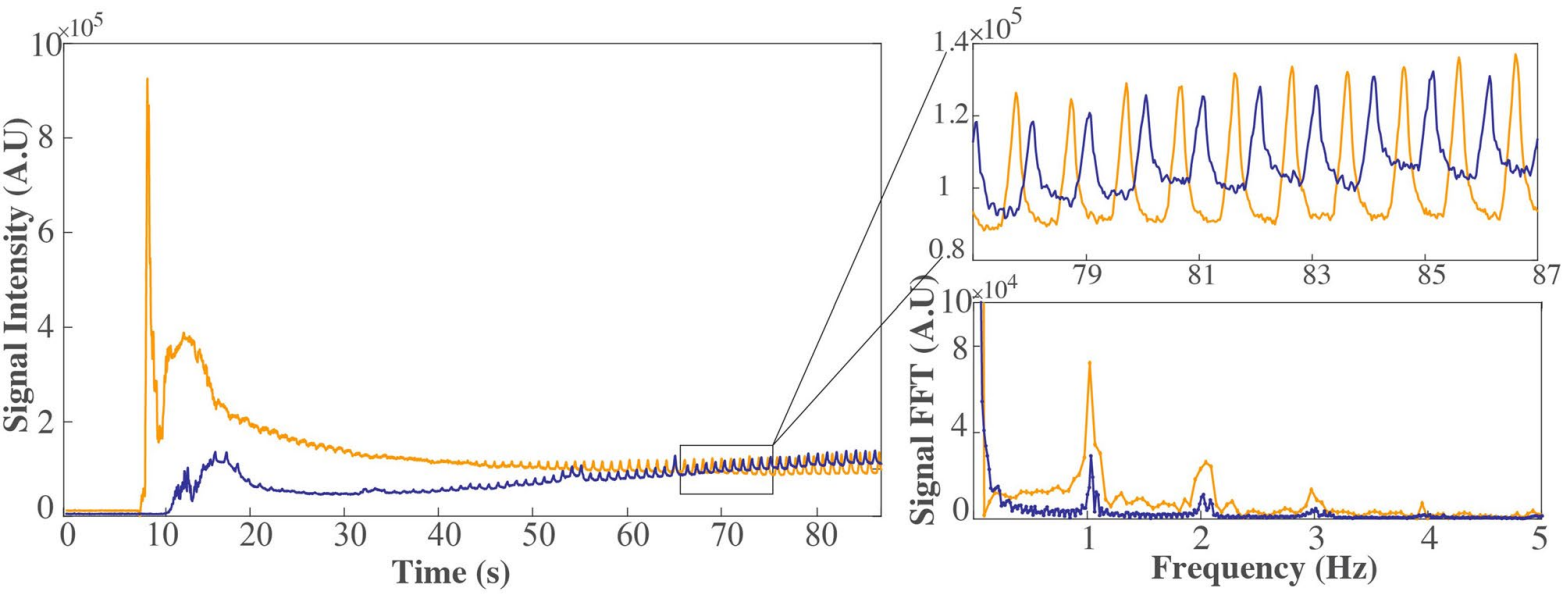
Biosignal derived from MPI signal*.* A signal component of the MPI measurement before background subtraction for both MNP—MCP 3 (orange) and Resovist (purple)—administered at a dose of 0.05 mmol Fe/kg. The modulation of the signal and the distinct peak in the Fourier transform of the signal correspond to a respiratory frequency of approximately 60 (breaths/minute).

A signal modulation is seen in the raw signal (Fig. 5). From the periodicity of the signal and/or its Fourier spectrum, distinct biological frequencies can be derived. The signal modulation is largely attributable to respiratory motion of the anesthetized rats. Based on a temporal resolution of 46 3D f/s a respiratory rate of approximately 60 (breaths/minute) can be estimated. Monitoring the signal modulation in raw signal also assured the clearance of both MNP types out of blood stream prior to the subsequent injection. The gained knowledge in combination with data from literature^7,15–18^ affirms that 1 h interval between the subsequent injections is sufficient not to influence the subsequent MPI scan.

## Discussion

MPI has potentially high sensitivity and high contrast. This combined with a high temporal resolution of 46 3D f/s makes MPI a promising technique for cardiovascular imaging. MPI physics relies on direct measurement of electronic magnetization of MNP, rather than nuclear magnetization as in MRI, hence a much higher sensitivity in detection of MNP in MPI is possible^19^. Former theoretical modeling studies predicted picogram sensitivity with MPI-optimal MNP^20^. In vitro data suggest that a MPI sensitivity below 100 ng can be achieved^6,21^. The former studies on in vivo vascular MPI with a spatial resolution in the millimeter range were performed with MNP dosages above the applied dosage in our study^2^. The newly developed MCP 3 indicates a better spatial resolution with at least 10-fold lower dosage applied, compared to conventional tracers used in angiographic system function (SF) based MPI studies (Resovist of 0.9 mmol Fe/kg^22^), and at least twofold lower dosage compared to the MPI-tailored MNP LS-008 (with approximately 0.15 mmol Fe/kg^7^) in mice. The applied dose is not far from the range of tolerable doses for MR clinical imaging with clinically approved iron oxide nanoparticles with maximum recommended Resovist doses of 0.45 mmol iron^23^. Moreover, in vitro studies have shown that submillimeter resolution is feasible by MPI-tailored MNP in conjunction with high-gradient amplitudes^21,24^. The development of MPI hardware and MPI-tailored MNP are necessary for further improvements of MPI towards clinical applications. However, hardware improvement is more challenging while optimization of MPI-tailored MNP could drastically reduce the costs of clinical MPI.

Furthermore, MCP 3-based MPI using relatively low tracer concentrations of 0.1 and 0.05 mmol Fe/kg allowed us to assess the arterial phase and to quantify aortic D_*L*_. Hence, we conclude that quantification of vascular abnormalities such as stenosis or aneurysm with high temporal resolution combined with quantitative measurement of tracer concentration is achievable by MPI. Additionally, real-time monitoring of vital physiological signs (respiratory and cardiac) is possible from the information derived from raw MPI signal, which can be beneficial in cardiovascular imaging. For instance, the derived biosignal could be of value for respiratory motion compensation.

Our study has some limitations. To date, MPI is limited in spatial resolution leading to partial volume effects and possible under- or overestimation of D_*L*_^8^. Additionally, the voxel dimension, independent of the spatial resolution of MNP, restrains the D_*L*_ measurement. Merely fractional amounts of signal in one neighboring voxel will result into significant deviation. The D_*L*_ measurement with MPI is relative. Similar to conventional CT-angiography, variations in reconstruction parameters can alter the D_*L*_ measurement. Standardized set of reconstruction parameters, based on previous phantom studies^14^ were applied for all examinations to maintain results comparable and reliable. Nevertheless, the obtained values of AA D_*L*_ and IVC D_*L*_ are in good agreement with the literature^25^. In future work, we will use co-registration with MRI for a more accurate localization of the vessels and thus more precise D_*L*_ quantification. The choice of Resovist (containing the drug substance Ferucarbotran) as a measure for comparison with MCP 3 is due to the frequent application of the tracer in MPI studies. Implementation of other commercially available traces with promising MPI characteristics such as perimag^26,27^ and synomag-D^28^ (micromod Partikeltechnologie GmbH, Rostock, Germany) and comparison with the obtained results would be of interest in future studies.

Another general limitation in MPI based on SF is the possible mismatch of the SF-sample used for calibration and the magnetization dynamics of MNP under influence of their specific local environment in vivo^29–32^, which causes image artifacts and reduced sensitivity of MPI^33^. In a further step, we will investigate to which extent the adaptation of the SF e.g. adaptation of the temperature between system function measurement and in-vivo imaging can improve the image quality.

## Conclusion

Our study demonstrates the feasibility of MPI angiographic imaging with potential quantitative assessment of vascular anatomy in vivo, which has been made possible by advancements in scanner hardware and new, more refined MCP 3 tracers. Collectively, these developments improved tracking of the intravenously injected bolus in the IVC, detection and differentiation of the first pass in the AA in a quantitative manner, and visualization of the morphology of large abdominal vessels and their branches. Using MCP 3-MPI vessel lumens of 2.7 ± 0.6 mm for IVC and 2.4 ± 0.7 mm for AA became detectable with a clinically acceptable dose of 0.05 mmol Fe/kg. Our study opens a window into higher resolution MPI for preclinical and clinical research.

## Materials and methods

### Tracers

Both tracers investigated and compared in this study are composed of magnetite/maghemite. They are synthesized by aqueous coprecipitation and have been proven biocompatible and biodegradable^16,23^.

MCP 3 was developed in-house as an MPI tracer coated with carboxymethyl dextran (CMD). The mean diameter of MCP cores is 32 nm (TEM) and the mean hydrodynamic diameter (D_H_) is 53 nm (DLS)^14^. The blood half-life of MCP in rats has been determined with MRI, 8.8 and 17.4 min at 0.05 and 0.1 mmol Fe/kg^16^.

Resovist (containing the drug substance Ferucarbotran), is an approved liver-specific MRI contrast agent (Schering AG, Berlin, Germany) that was taken off the market in Europe in 2008 but is still available in Japan (Fujifilm RI Pharma, Tokyo, Japan). It is coated with carboxydextran (CDX) and has a bimodal size distribution with mean core diameters of about 4 nm and 16 nm (TEM)^34,35^. The particles have a mean hydrodynamic diameter (D_H_) of 60 nm (DLS)^36^ and has a blood half-life of 3.9–5.8 min in MRI^18^ and less than 15 min in MPI^7,15,17^.

### Imaging hardware and image reconstruction

Images were acquired in a preclinical 25/20 FF MPI scanner (Bruker Biospin/Philips, Germany) equipped with a separate receive-only gradiometer coil for increased sensitivity^6^. The acquisition was performed simultaneously with both the preinstalled coil and the separate receive coil. The 25/20 FF MPI scanner is a field-free-point (FFP)-based system and requires a SF calibration measurement (Supplement S3) for image reconstruction^37,38^. The nanoparticles were excited by drive fields of 12 mT in three orthogonal axes at three slightly different frequencies (2.5 MHz divided by 102/96/99 in x-/y-/z-direction). A selection gradient with a strength of 1.25 T/m/µ_0_ in x- and y-axis and 2.5 T/m/µ_0_ in z-axis is used for spatial encoding and generates the FFP. The FFP is moved along a 3D-Lissajous trajectory by the three drive fields and scans the field of view (FOV) of 19.2 × 19.2 × 9.6 mm^3^ in 21.54 ms, resulting in 46 frames/s. The acquired data were reconstructed via the Kaczmarz algorithm with Tikhonov regularization in ParaVision 6 MPI software (Bruker Biospin, Ettlingen, Germany) to 33 × 33 × 33 voxels covering a volume of 26.4 × 26.4 × 13.2 mm^3^ leading to an overscan of the FOV^39^ to avoid artifacts at the borders. For further details of image reconstruction see supplement (Supplement S4).

### Image analysis

Three-dimensional (3D) images were visualized and analyzed in MATLAB (2016a, Mathworks, Natick, MA, USA). For quantitative analysis, iron concentration–time curves were generated. A 3D VOI on the cross-section of the IVC and AA was defined, and mean signal in the VOI was determined throughout image acquisition. Digital subtraction of the IVC was performed in temporally separated images, a single frame during bolus passage in the IVC (t_1_) (Fig. 1), to separate arterial phase in the AA from the adjacent IVC in images. D_*L*_ of the IVC and AA were determined from separated vessels in images. To this end, vessel cross-sections were segmented in two-dimensional planes using basic intensity-based segmentation with a cutoff threshold of 30% maximum intensity^14^. The cross-sectional area was calculated as the sum of the number of all pixels in the segmented area multiplied by voxel size in transverse direction. Assuming the cross-section is circular in the transverse plane, the D_*L*_ was calculated from the area. The image quality was assessed using visual grading analysis (VGA) including visual grading analysis score (VGAS). The image criteria identified for a score scale are listed in Table 1. The overall score was defined as sum of all VGAS attributed to one examination. The statistical difference was analyzed by Mann–Whitney U-test using GraphPad Prism software version 7 (GraphPad Software, San Diego, CA, USA) and differences were considered significant for P ≤ 0.05.

**Table 1:** The image quality criteria and the score scale defined to evaluate the quality of images.

Score Criterion	1	2	3	4
IVC visibility	Not visible	Poorly visible	Visible	Very well visible
AA visibility	Not visible	Poorly visible	Visible	Very well visible
Background (BG) artifact	Extensive artifact	Moderate artifact	Minimal artifact	No artifact

The image quality was graded visually. A higher score corresponds to better image quality and vice versa.

### Animal experiments

**Figure 6:**
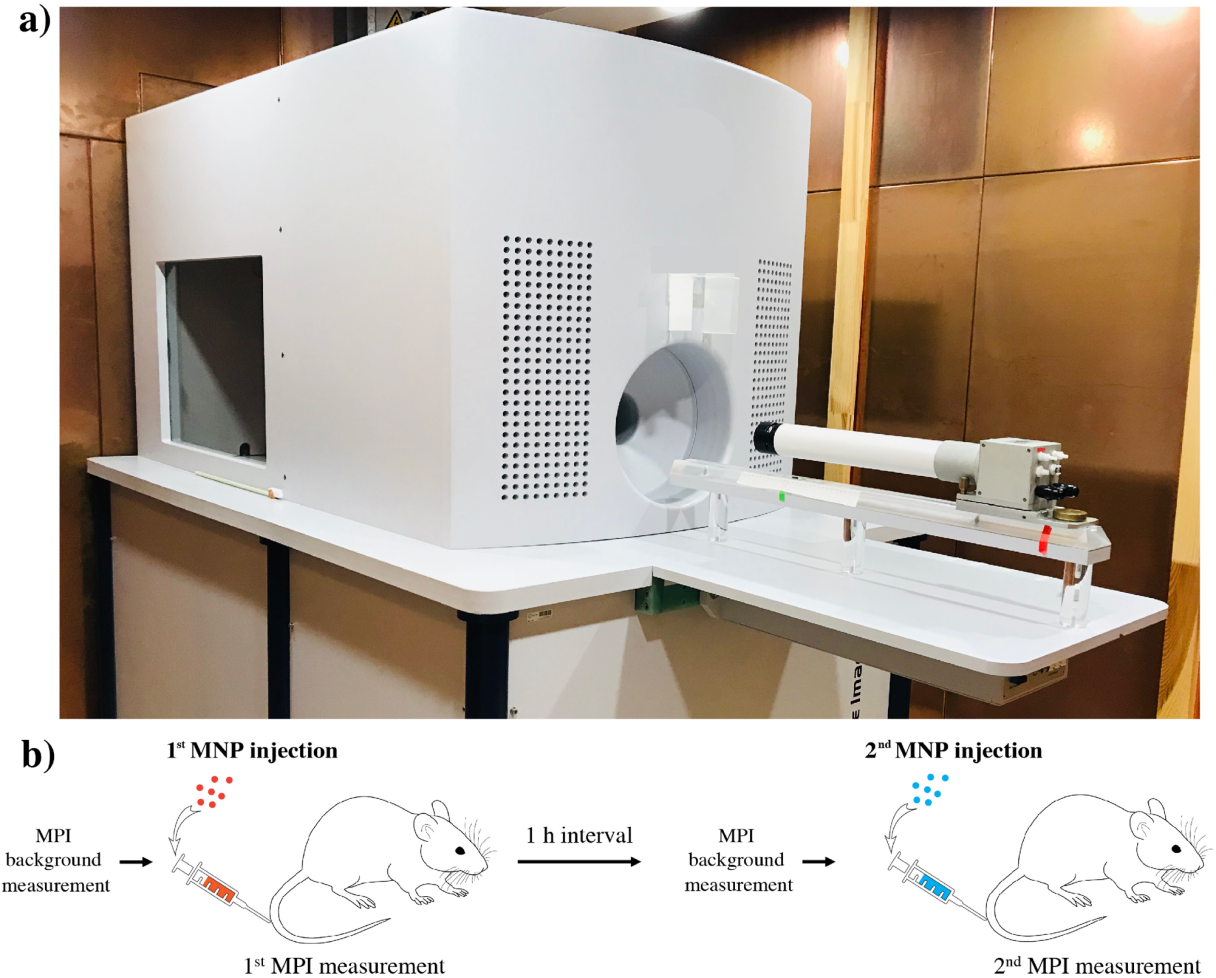
Preclinical MPI scanner **(a)** and diagram of experimental workflow **(b)**. **(a)** Preclinical MPI scanner (MPI 25/20 FF, Bruker BioSpin) in a copper-shielded cabinet to suppress interferences from external electromagnetic fields. **(b)** Diagram of experimental workflow. First, background MPI signal is acquired, followed by IV injection of one of the MNP and MPI scanning for several minutes. After 1 h (no signals from MNP detectable in blood vessels), the second tracer, was injected intravenously. The order of MNP type administration was random.

All animal studies were approved by the State Office of Health and Social Affairs Berlin (LAGESO/A0409/12) and were carried out in accordance with institutional and federal animal care guidelines. Overall, 16 in vivo examinations were carried out on 8 healthy male Sprague Dawley rats (Charles River Laboratories, Sulzfeld, Germany), 7 weeks old and had an average body weight of 259 ± 19 g. Prior to MPI scan, rats were anesthetized in an anaesthetic induction chamber with 5% isoflurane and maintained in 1–2% isoflurane during MPI acquisitions. Each rat was imaged twice, once with MCP 3 and once with Resovist. The order of MNP type administration was random. Identical doses of MCP 3 and Resovist were successively administered intravenously (Fig. 6) with sufficient time between the examinations for clearance of the MNP from the blood (at least 1 h), when no signal modulation was detected prior to the MPI scans, as seen in raw data on the baseline signal (Fig. 5). The concentrations of the stock solutions were 145.5 mmol Fe/l for MCP 3 and 500 mmol Fe/l for Resovist, and the final administered doses were 0.025, 0.5, and 0.1 mmol Fe/kg of bodyweight. Final concentrations were prepared by diluting stock solutions with 7.5% mannitol. MPI acquisition, duration 2–5 min, started approximately 1 min before injection.

### Compliance with ethical standards

All animal studies were approved by the State Office of Health and Social Affairs Berlin (LAGESO) and were carried out in accordance with institutional and federal animal care guidelines.

## Supplementary information

Supplementary Information.

## Abbreviations

AA: Abdominal aorta
CDX: Carboxydextran
CMD: Carboxymethyl dextran
D_*L*_: Lumen diameter
DLS: Dynamic light scattering
FFP: Field-free point
FOV: Field of view
IVC: Inferior vena cava
MCP 3: Multicore nanoparticles
MNP: Magnetic nanoparticle
MPI: Magnetic particle imaging
SF: System function
TEM: Transmission electron microscopy
VGA: Visual grading analysis
VOI: Volume of interest
