# Supplementary material for: In vivo magnetic particle imaging: angiography of inferior vena cava and aorta in rats using newly developed multicore particles
Source: Sci Rep. 2020 Oct 14;10:17247. doi: 10.1038/s41598-020-74151-4 (PMC7560824; doi:10.1038/s41598-020-74151-4)
Supplement: Supplementary file 1 — Supplementary Information. [file 41598_2020_74151_MOESM1_ESM.docx]

**Title Page**

# “In Vivo Magnetic Particle Imaging: Angiography of Inferior Vena Cava and Aorta in Rats Using Newly Developed Multicore Particles”

**List of authors:**

Azadeh Mohtashamdolatshahi^1*^

Harald Kratz^1^

Olaf Kosch^2^

Ralf Hauptmann^1^

Nicola Stolzenburg^1^

Frank Wiekhorst^2^

Ingolf Sack^1^

Bernd Hamm^1^

Matthias Taupitz^1^

Jörg Schnorr^1^

**Affiliations:**

^1^ Charité - Universitätsmedizin Berlin, corporate member of Freie Universität Berlin, Humboldt-Universität zu Berlin, and Berlin Institute of Health, Department of Radiology, 10117 Berlin, Germany

^2^Department of Medical Physics and Metrological Information Technology, Physikalisch-Technische Bundesanstalt (PTB), Abbestrasse 2-12, 10587 Berlin, Germany

**Short title for running head:** “In Vivo Magnetic Particle Imaging: Angiography”

**Corresponding author:**

Azadeh Mohtashamdolatshahi

Experimental Radiology

Institute for Radiology, Charite – University of Medicine Berlin

Chariteplatz 1, 10117, Berlin

Office: +49 30/450 539074

Fax: +49 30/450-539 901

E-Mail: Azadeh.Mohtashamdolatshahi@charite.de

**S1. IVC image at pre-,post- and peak time of bolus
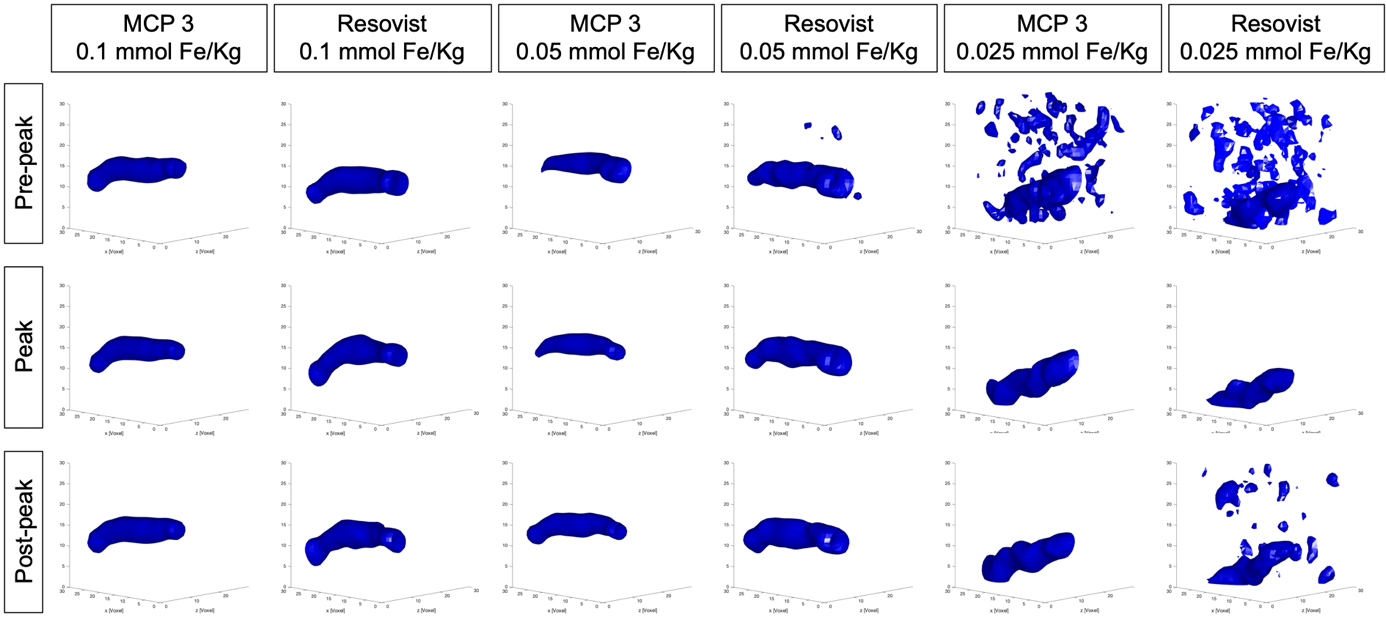
**

S1. Comparison of images of IVC at the time of peak, pre- and post- peak. The images of IVC at the peak time of MNP arrival and a short time period after provide similar adequate image quality.

**S2. MPI spectra of MCP 3 and Resovist**


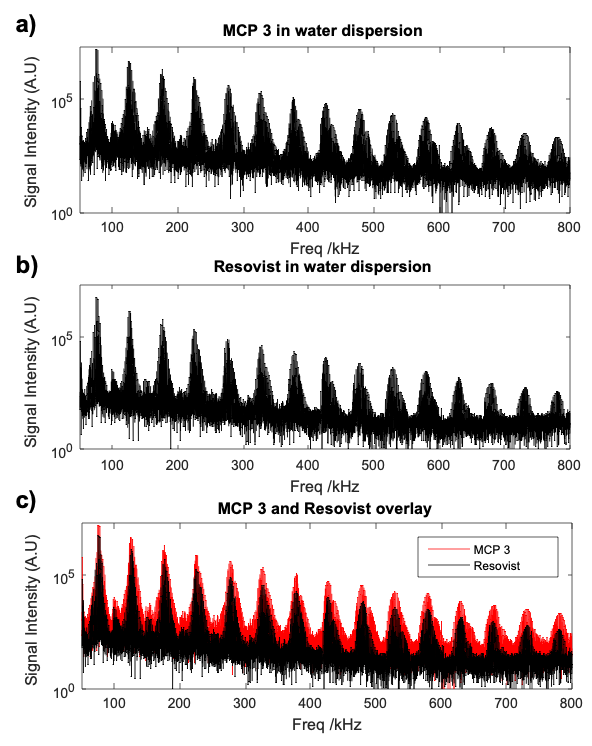


S2. MPI spectra of MNPs normalized to the iron amount a) MCP 3 in water dispersion b) Resovist in water dispersion c) Overlay of both spectra. MCP 3 provides a greater signal amplitude and a slower decay of higher harmonics than Resovist.

**S3. System Functions measurements**

The SF measurement was performed prior to image reconstruction. The delta point samples of each tracer had a volume of 4 µl tracer water dispersed in a PCR tube which estimates to a dimension of 2 x 2 x 1 mm^3^. The SF samples had a concentration of 100 mmol/l for Resovist and 70 mmol/l for MCP. A robot arm moved and positioned the sample in center of grid points (33x33x33). The SF measurements were acquired with the same drive and gradient fields aforementioned.

**S4. Image Reconstruction**

The acquired data were reconstructed to 33x33x33 voxels via Kaczmarz’s algorithm with Tikhonov regularization in ParaVision 6 MPI software (Bruker Biospin, Ettlingen, Germany). The hardware background noise limits the used frequency components to a bandwidth to 0.09-125 MHz so the 3^rd^ harmonic is as well filtered out. The acquired images were reconstructed once with frequencies obtained with preinstalled coil in x and y channel, and once with the frequencies obtained with the Rx coil. For both MCP 3 and Resovist MNPs 2501 frequencies were chosen automatically according to the applied system function and the SNR threshold based on SF. The order of mixing the frequencies for all the images was 25. Prior to reconstruction the background signal for the same time interval as the measurement was subtracted. For reconstruction, a moving average of 5 repetitions was applied to the measurement to reduce visible noise in the images. The reconstruction parameter, regularization factor (λ) was chosen based on our previous work, where the SNR was at highest in phantom studies with 5 iterations. These parameters such were kept constant in all reconstructions to exclude the influence of variations in these parameters to maintain results comparable.
